# Clinical efficacy and biomarker analysis of neoadjuvant camrelizumab plus chemotherapy for early-stage triple-negative breast cancer: a experimental single-arm phase II clinical trial pilot study

**DOI:** 10.1097/JS9.0000000000001011

**Published:** 2023-12-19

**Authors:** Chunhui Zheng, Yanbing Liu, Xue’er Wang, Zhao Bi, Pengfei Qiu, Guangdong Qiao, Xiang Bi, Zhiqiang Shi, Zhaopeng Zhang, Peng Chen, Xiao Sun, Chunjian Wang, Shiguang Zhu, Xiangjing Meng, Yunjie Song, Yingxue Qi, Lu Li, Ningning Luo, Yongsheng Wang

**Affiliations:** a Breast Cancer Center, Shandong Cancer Hospital and Institute, Shandong First Medical University and Shandong Academy of Medical Sciences, Jinan; b Tianjin Medical University Cancer Institute & Hospital, National Clinical Research Center for Cancer, Key Laboratory of Cancer Prevention and Therapy, Tianjin, Tianjin’s Clinical Research Center for Cancer, Tianjin; c Breast Cancer Center, The Affiliated Yantai Yuhuangding Hospital of Qingdao University, Yantai; d Toxicology Research Center, Shandong Academy of Occupational Health and Occupational Medicine, Shandong First Medical University & Shandong Academy of Medical Sciences, Jinan; e Jiangsu Simcere Diagnostics Co., Ltd., Nanjing Simcere Medical Laboratory Science Co., Ltd., The State Key Laboratory of Neurology and Oncology Drug Developmen, Nanjing, China

**Keywords:** camrelizumab, immunotherapy, neoadjuvant therapy, triple-negative breast cancer, tumor immune microenvironment

## Abstract

**Background:**

Triple-negative breast cancer (TNBC) is associated with a dismal prognosis. Immune checkpoint inhibitors have shown promising antitumor activity in neoadjuvant settings. This single-arm, phase II trial aimed to evaluate the efficacy and safety of camrelizumab plus chemotherapy as the neoadjuvant therapy (NAT) in early TNBC.

**Methods:**

Patients received eight cycles of camrelizumab plus nonplatinum-based chemotherapy. The primary endpoint was total pathological complete response (pCR). Secondary endpoints included the breast pathological complete response (bpCR), adverse events (AEs). Multiomics biomarkers were assessed as exploratory objective.

**Results:**

Twenty of 23 TNBC patients receiving NAT underwent surgery, with the total pCR rate of 65% (13/20) and bpCR rate of 70% (14/20). Grade ≥3 treatment-related AEs were observed in 14 (60.9%) patients, with the most common AE being neutropenia (65.2%). Tumor immune microenvironment was analyzed between pCR and non-pCR samples before and after the NAT. Gene expression profiling showed a higher immune infiltration in pCR patients than non-pCR patients in pre-NAT samples. Through establishment of a predictive model for the NAT efficacy, *TAP1* and *IRF4* were identified as the potential predictive biomarkers for response to the NAT. Gene set enrichment analysis revealed the glycolysis and hypoxia pathways were significantly activated in non-pCR patients before the NAT, and this hypoxia was aggravated after the NAT.

**Conclusion:**

Camrelizumab plus nonplatinum-based chemotherapy shows a promising pCR rate in early-stage TNBC, with an acceptable safety profile. *TAP1* and *IRF4* may serve as potential predictive biomarkers for response to the NAT. Aggravated hypoxia and activated glycolysis after the NAT may be associated with the treatment resistance.

## Introduction

Highlights

- Neoadjuvant camrelizumab plus nonplatinum-based chemotherapy shows a high pathological complete response rate in triple-negative breast cancer.
- *TAP1* and *IRF4* may be potential predictive biomarkers for response to the neoadjuvant therapy.
- Aggravated hypoxia and activated glycolysis after the neoadjuvant therapy may be associated with treatment resistance.

Triple-negative breast cancer (TNBC) is associated with aggressive tumor biology and a dismal prognosis, approximately accounting for 15% of invasive breast cancers^1^. Currently, neoadjuvant therapy (NAT) is the preferred option for high-risk early TNBC^2^, contributing to increased operability and eligibility for breast-conserving surgery (BCS). Furthermore, surgical outcomes may identify the patients who can avoid subsequent therapies, and patients who achieve pathological complete response (pCR) after NAT have favorable prognosis. Therefore, efforts have been made to develop novel therapeutic strategies to improve pCR rates of TNBC.

Recently, numerous trials have shown that addition of immune checkpoint inhibitors to the NAT can increase pCR rates^3–5^. However, novel neoadjuvant modalities for TNBC are limited. Anthracycline-based and taxane-based chemotherapy is considered as the first-level NAT regimen for localized TNBC^6^. Adding platinum agents to this NAT regimen can improve the pCR rates of TNBC, but it can also increase toxicity^7^. A phase III trial (KEYNOTE-522) showed that adding pembrolizumab to platinum-containing chemotherapy could improve the pCR rates for TNBC patients^3^. Camrelizumab, an IgG4-k PD-1 monoclonal antibody, has shown effectiveness and economy in China^8,9^. However, there are few studies reporting its use in the neoadjuvant setting of TNBC.

Immunologic parameters have been shown to affect the response to immune checkpoint inhibitors^10^. Here, we conducted a phase II study to evaluate the efficacy and safety of camrelizumab combined with platinum-free chemotherapy as the NAT in patients with early TNBC, and evaluated the predictive value of immune-related gene expressions in the chemoimmunotherapy response^11^.

## Methods

### Study design and patients

This was an open-label, single-arm, phase II trial. Women were eligible for enrollment if they were at the age of greater than or equal to 18 years and had an Eastern Cooperative Oncology Group (ECOG) performance status score of 0 or 1; adequate organ function; histologically confirmed noninflammatory invasive TNBC defined as estrogen receptor/progesterone receptor (ER/PR)-negative (ER/PR staining of <1%) and human epidermal growth factor receptor 2 (HER2)/Neu-negative [immunohistochemistry (IHC) 0-1+ or IHC 2+ and chromogenic/fluorescent in situ hybridization negative] based on the guidelines of the American Society of Clinical Oncology-College of American Pathologists; stages II–III (T1cN1-2 or T2-4N0-2) disease with surgically resectable lesions. Patients were excluded if they had metastatic diseases, autoimmune diseases, history of concurrent malignancies, or history of severe allergic reactions to monoclonal antibodies, received anticancer treatment, or immunosuppressive therapy. The work has been reported in line with the strengthening the reporting of cohort, cross-sectional and case–control studies in surgery (STROCSS) criteria^11^ (Supplemental Digital Content 1, http://links.lww.com/JS9/B610).

### Treatment

**Figure 1:**
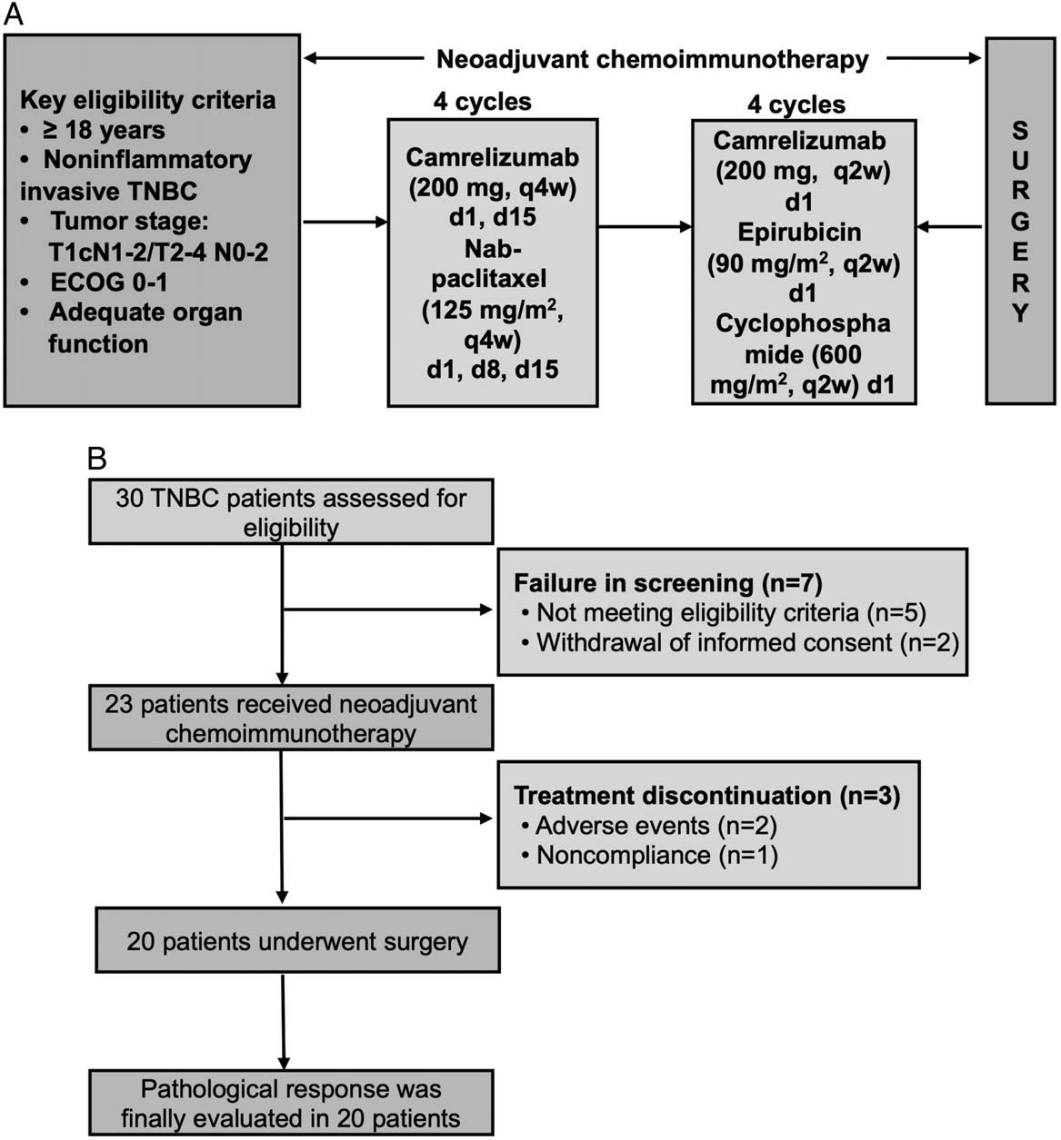
Study design. (A) The flowchart of neoadjuvant therapy. (B) The flowchart of enrolled patients.

Patients received four cycles of camrelizumab (200 mg, d1, d15, q4w) plus nab-paclitaxel (125 mg/m^2^, d1, d8, d15, q4w), and four cycles of camrelizumab (200 mg, d1, q2w) plus epirubicin (90 mg/m^2^, d1, q2w) + cyclophosphamide (600 mg/m^2^, d1, q2w). Surgery was performed 2~4 weeks after the last cycle of NAT (Figure 1A). The surgical types, such as mastectomy or BCS, sentinel lymph node biopsy or axillary lymph node dissection, were determined by the surgeon according to the radiological response. Adjuvant treatment was decided by a multidisciplinary team based on the pathologic response. All patients received camrelizumab once every 3 weeks for up to nine cycles after surgery. Of note, adjuvant camrelizumab plus capecitabine regimen was recommended for non-pCR patients.

Patients were restaged following baseline imaging, including breast MRI, computed tomography scanning for chest and abdomen and/or abdominal sonography, and radionuclide bone imaging. Follow-up and imaging were planned for every 2 weeks. Blood chemistry analyses were done for every cycle. The dose of chemotherapy agents was reduced due to certain adverse events (AEs).

### Endpoints and assessments

The primary endpoint was the total pCR, defined as absence of invasive tumor cells in the breast and axilla (ypT0/is ypN0) in the resected specimen. The resected specimens were independently assessed by two experienced pathologists. Secondary endpoints included the breast pathological complete response (bpCR, ypT0/is) and AEs during the NAT period which were collected following the National Cancer Institute Common Terminology Criteria for Adverse Events (version 4.0). The exploratory endpoint included mutational analysis of capture-based targeted deep sequencing and tumor immune microenvironment (TIME) analysis of the gene expression profiling.

### Mutational analysis

Mutational analysis of tumor samples was assessed by performing capture-based targeted deep sequencing using FoundationOne CDx assay^12^, which could sequence the complete exons of 324 cancer-related genes and detect substitutions, insertions and deletions (indels), copy number variations (CNVs) and gene rearrangements. DNA extraction, library construction, sequencing, and bioinformatics analysis were all assessed based on standard methods, as previously described^13^.

### RNA expression detecting

Gene expression profiling of 770 immune-related genes (including 20 housekeeping genes) was performed using the Nanostring nCounter PanCancer IO360 Panel (NanoString Technologies)^14^. Ten unstained slides of 5 µm thickness were obtained from Formalin-fixed paraffin-embedded blocks. RNA expression detecting proceed according to the NanoString tech note (Panel Standard and Calibration Sample Usage). Normalized counts were finally log2 transformed for downstream analysis.

### Differentially expressed genes (DEGs) and signal pathway enrichment analysis

The DESeq2 package of R was used to perform the DEGs analysis. The *P*-value threshold was determined by controlling the false discovery rate (FDR) with the Benjamini algorithm. Genes with FDR less than 0.05 were identified to be differentially expressed, and then gene set enrichment analysis (GSEA) analysis was performed. The Hallmark and gene ontology gene sets were obtained from the Molecular Signatures Database (MSigDB). The volcano and heatmap plots for DEGs were visualized by the ggplot2 package (3.3.3).

### Analysis of TIME characteristics

Marker genes of TIME characteristics were retrieved as previously reported^15^. The arithmetic mean value of genes involved in all immune cell types was calculated as the score of cell type indicators, and Wilcoxon test was used to identify the difference between different groups.

### Statistical analysis

Based on patient characteristics and drug accessibility, combined with previously published research results that the pCR rate after anthracycline-based and taxane-based NAT was 30%^16^. We expected that camrelizumab added to chemotherapy would increase the pCR rate to 65%. Eleven patients would provide at least 80% power to detect the difference with one-sided α of 0.05, considering a drop-out of 10%, a total of 13 patients was required.

R software (version 3.6.1) was employed for statistical analysis. Differences between groups were evaluated using Wilcoxon rank-sum tests for continuous data and Fisher’s exact tests for categorical variables. Pearson’s test was used for the correlation analysis. Calculation of the area under the receiver operating characteristic curve was used as a measure of discriminatory ability for the signature scores. The value of *P*<0.05 was considered statistically significant.

## Results

### Patient characteristics

From June 2020 to August 2021, 30 patients were assessed for eligibility, among whom five were unqualified for eligibility criteria and two withdrew informed consent. Finally, 23 patients were enrolled and received NAT (Figure 1B)). Their median age was 52 years old. Most patients had the tumor stage of T2 (87.0%), positive nodal involvement (69.6%), stage II disease (73.9%), and ECOG performance status score of 0 (87.0%). Detailed demographics are shown in Table 1.

**Table 1:** Baseline characteristics of 23 patients with TNBC, *n* (%).

Characteristics	Eligible patients
Median age (range), years	52 (29–65)
T stage	
T1	2 (8.7)
T2	20 (87.0)
T3	1 (4.3)
Nodal status	
Positive	16 (69.6)
Negative	7 (30.4)
Clinical stage	
II	17 (73.9)
III	6 (26.1)
ECOG performance status	
0	20 (87.0)
1	2 (8.7)
Unknown	1 (4.3)

ECOG, Eastern cooperative oncology group; TNBC, triple-negative breast cancer.

During the NAT period, 2 (8.7%) patients discontinued treatment due to unacceptable AEs, and 1 (4.3%) was excluded due to noncompliance. None of them received surgery. Ultimately, 20 patients underwent surgery and pathological responses were evaluated (Figure 1B).

### Treatment response

**Figure 2:**
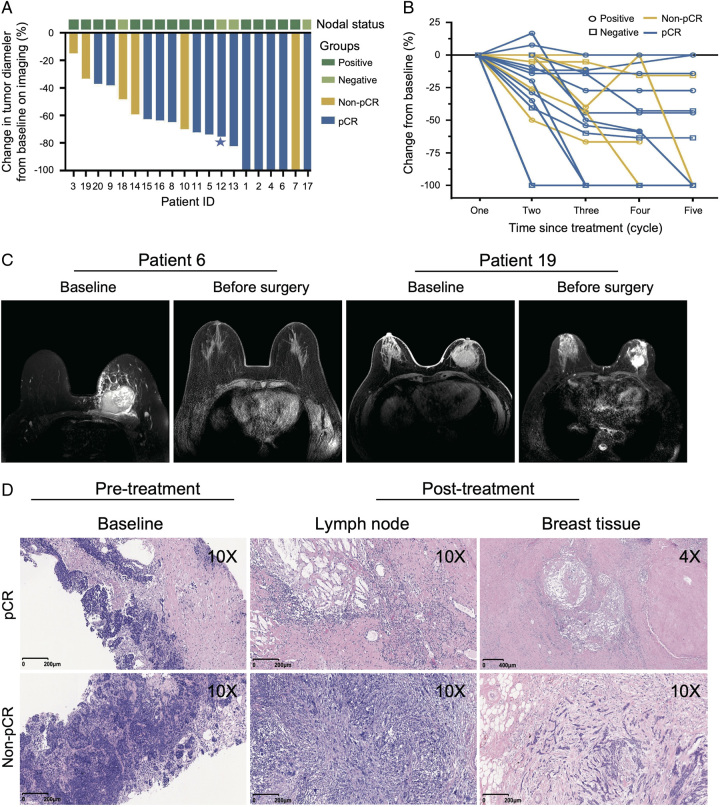
Radiological and pathological responses to neoadjuvant camrelizumab combined with chemotherapy. (A) Waterfall plots of the best radiological response based on RECIST V1.1. (B) Changes in tumor burden from the baseline in the efficacy-evaluable population from one to five cycles (*n*=20). We found no prominent changes from five to eight cycles (date not shown). (C) Radiological responses of representative patients with pCR before and after the neoadjuvant therapy. The breast tumor showed significant shrinkage after the neoadjuvant therapy. D) H&E images of pCR and non-pCR patients before and after the neoadjuvant therapy. pCR, pathological complete response; RECIST, response evaluation criteria in solid tumors.

For 20 patients undergoing surgery, 13 (65.0%) achieved total pCR, and 14 (70.0%) achieved bpCR (Figure 2A). Notably, 8 (57.1%) did not obtain CR radiologically but showed a marked tumor regression pathologically at surgery. For 16 cases of positive axillary lymph nodes, 11 (68.7%) achieved bpCR. Figure 2B describes the changes of pathological responses after the NAT. Representative images of radiographical and pathological responses before and after the NAT are shown in (Figure 2C, D).

### AEs

Twenty of 23 (87.0%) patients completed the planned NAT, and 22 (95.7%) experienced at least one treatment-associated AEs. The most common AEs of any grade included leucopenia (95.7%), neutropenia (91.3%), nausea (78.3%), asthenia (65.2%), alopecia (65.2%), and more. Grade ≥3 treatment-related AEs were observed in 14 (60.9%) patients, with the most common AE being neutropenia (65.2%). Reactive cutaneous capillary endothelial proliferation (60.9%) was the most frequent immune-related AEs, followed by hypothyroidism (26.1%), hyperthyroidism (8.7%), pneumonia (8.7%), and more. Two patients (8.7%) experienced grade 4 pneumonia. Detailed treatment-related AEs are shown in Table 2.

**Table 2:** Treatment-related adverse events during neoadjuvant chemoimmunotherapy.

	Grades, *n* (%)		
Treatment-related adverse events	Any grade	Grade 3	Grade 4
Leucopenia	22 (95.7)	10 (43.5)	2 (8.7)
Neutropenia	21 (91.3)	9 (39.1)	6 (26.1)
Nausea	18 (78.3)	0	0
Asthenia	15 (65.2)	1 (4.3)	0
Alopecia	15 (65.2)	0	0
Anemia	14 (60.9)	2 (8.7)	0
Reactive cutaneous capillary endothelial proliferation	13 (56.5)	0	
Vomiting	13 (56.5)	0	0
Increased alanine aminotransferase	9 (39.1)	3 (13.0)	0
Hypoaesthesia	9 (39.1)	0	0
Increased aspartate aminotransferase	8 (34.8)	3 (13.0)	0
Diarrhea	7 (30.4)	0	0
Decreased lymphocyte count	7 (30.4)	3 (13.0)	1 (4.3)
Rash	7 (30.4)	0	0
Limb pain	7 (30.4)	0	0
Abdominal pain	5 (21.7)	0	0
Poor appetite	5 (21.7)	1 (4.3)	0
Immune-mediated adverse events			
Immune-related pneumonia	2 (8.7)	0 (0)	2 (8.7)
RCCEP	14 (60.9)	0 (0)	0 (0)
Hypothyroidism	6 (26.1)	0 (0)	0 (0)
Hyperthyroidism	2 (8.7)	0 (0)	0 (0)
Protein in urine	1 (4.3)	0 (0)	0 (0)
Oral mucositis	1 (4.3)	0 (0)	0 (0)

RCCEP, reactive cutaneous capillary endothelial proliferation; SEA, serious adverse events.

### Genomic landscape

**Figure 3:**
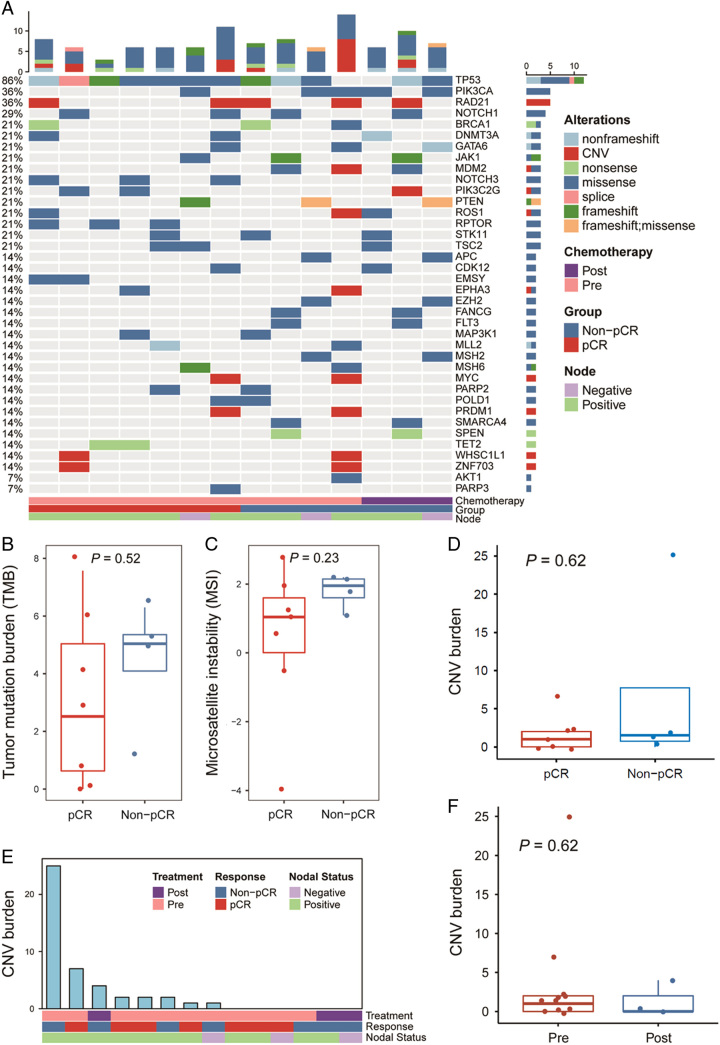
Mutational analysis before and after neoadjuvant camrelizumab combined with chemotherapy. (A) Genomic landscape before and after the neoadjuvant therapy, clinical and pathological data are displayed below variants. Columns represent individuals. Rows represent specific genes. (B–D) Box plots of the tumor mutational burden, microsatellite instability (mutations per megabase), and CNV burden in pCR (*n*=7) and non-pCR patients (*n*=4). (E) The general view of CNV burden. F) Changes of CNV burden before and after the neoadjuvant therapy. CNV, copy number variants; pCR, pathological complete response.

Of 11 patients sequenced using FoundationOne CDx assay, the frequently altered genes in TNBC were *TP53* (86.0%), followed by *PIK3CA* (36.0%), *RAD21* (36.0%), and *NOTCH1* (29.0%). *NOTCH3* and *RPTOR* mutations occurred in 43% (3/7) of the baseline pCR samples, but in 0 of non-pCR samples (0/4), possibly indicating a trend of good response (Figure 3A). However, no significant differences were presented between pCR and non-pCR patients in TMB, MSI, and CNV burden (Figure 3B–D). Additionally, no significant differences were seen in CNV burden before and after the NAT (Figure 3E, F).

### TIME between pCR and non-pCR samples

**Figure 4:**
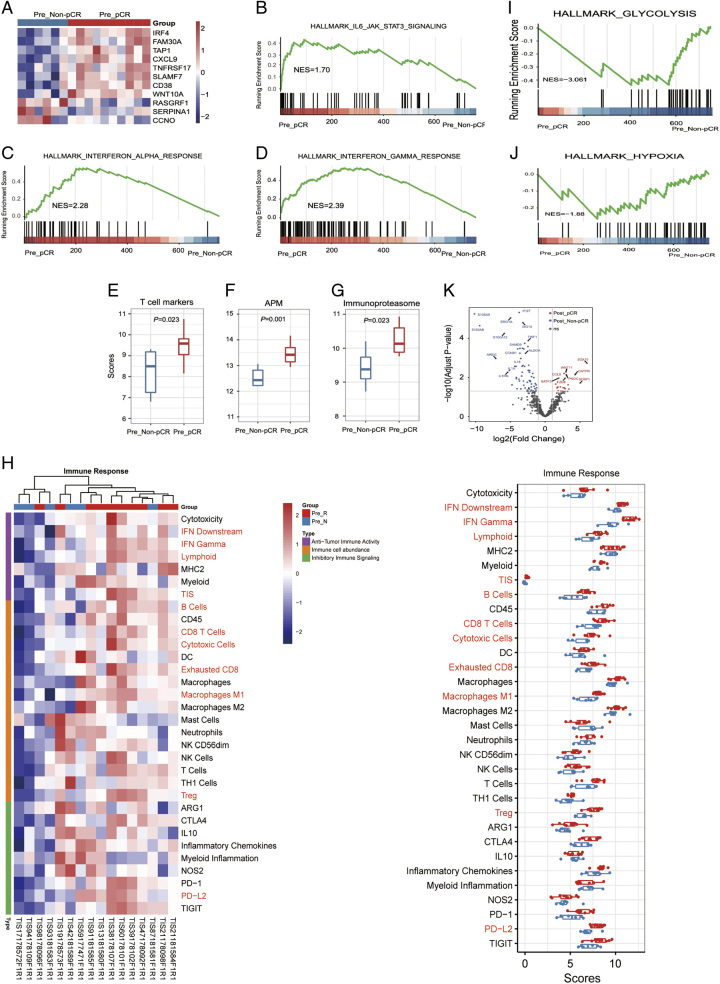
The tumor immune microenvironment of pCR and non-pCR patients before neoadjuvant therapy. (A) A waterfall plot of differentially expressed genes between pCR and non-pCR patients. (B–D) Gene set enrichment analysis of the Hallmark IL-6/JAK/STAT3 signaling, IFN-alpha, and IFN-gamma response pathways. (E–G) Comparison of the scores of T cell markers, APM and immunoproteasome between pCR and non-pCR patients. (H) A heatmap of immune responses between pCR and non-pCR patients. (I–J) Gene set enrichment analysis of the Hallmark glycolysis and hypoxia pathways between pCR and non-pCR patients before the neoadjuvant therapy. (K) The postneoadjuvant transcriptomic characteristics between pCR and non-pCR patients.

By comparison to the preneoadjuvant baseline information of pCR patients (*n*=10) with non-pCR patients (*n*=6), it was found no significant differences in the age, T stage, nodal status, clinical stage, and TNM stage (*P*>0.05; Table S1). We identified 11 DEGs, including eight up-regulated genes and three down-regulated genes in pre-pCR group (Figure 4A). Meanwhile, IL-6/JAK/STAT3 signaling, IFN-alpha, IFN-gamma, allograft rejection, and UV response pathways were significantly activated in pre-pCR group (Figure 4B–D; Supplementary Figure 1A, B, Supplemental Digital Content 2, http://links.lww.com/JS9/B611).

We further compared TIME-related indicators between pre-pCR and pre-non-pCR groups. The results showed that pre-pCR group had higher scores of T cell markers (*P*=0.023), antigen presentation machinery (*P*=0.001), immunoproteasome (*P*=0.023) (Figure 4E–G), and other characteristics of TIME (Figure 4H) than pre-non-pCR group. These findings highlighted a higher immune infiltration in pre-pCR group. GSEA analysis of pre-NAT revealed that glycolysis and hypoxia pathways were activated in pre-non-pCR group (Figure 4I–J).

The postneoadjuvant transcriptomic characteristics between pCR and non-pCR patients were compared, and 19 up-regulated genes and 62 down-regulated genes in pCR patients were identified (Figure 4K) and we found that the tumors proliferated significantly in post-non-pCR group than post-pCR group (*P*=0.0095; Supplementary Figure 1F, Supplemental Digital Content 2, http://links.lww.com/JS9/B611).

### Construction of predictive model for the NAT efficacy

**Figure 5:**
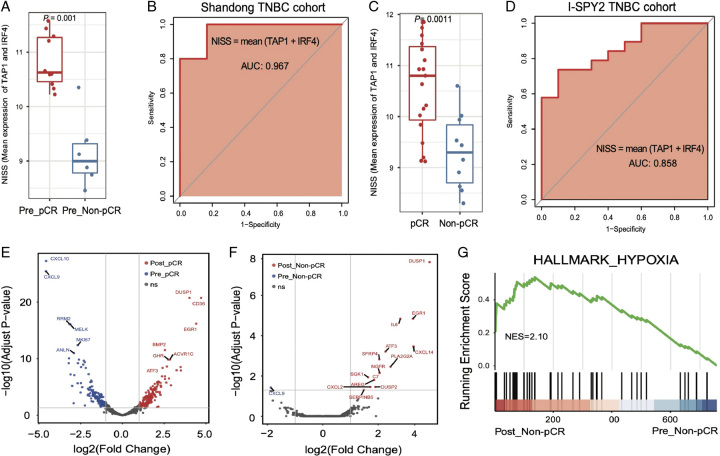
Dynamic changes of tumor immune microenvironment before and after the neoadjuvant therapy. (A–B) Comparison of the NISS between pCR and non-pCR patients, and the receiver operator characteristic curve of the NISS in our cohort. (C–D) Comparison of the NISS between pCR and non-pCR patients, and the receiver operator characteristic curve of the NISS in I-SPY2- TNBC cohort. (E–F) The preneoadjuvant and postneoadjuvant transcriptomic characteristics in pCR and non-pCR patients. (G) Gene set enrichment analysis of the hypoxia pathway in non-pCR patients after the neoadjuvant therapy.

Then 11 DEGs between pre-pCR and pre-non-pCR groups and 148 genes involved in TIME characteristics with significant differences were analyzed, and seven genes were the intersection of the two. These seven genes were used to establish the single-gene predictive model for the NAT efficacy (Supplementary Figure 1C, Supplemental Digital Content 2, http://links.lww.com/JS9/B611). The final predictive model [neoadjuvant immunotherapy sensitivity score (NISS)] was built using the genes with the predictive capability more than 0.9, namely the mean expression of TAP1 plus IRF4, which showed a significant difference between pre-pCR and pre-non-pCR groups (*P*=0.001; Figure 5A). In our cohort, the NISS presented an area under the curve (AUC) of 0.967 (Figure 5B). According to the optimal cutoff value, the binary threshold was determined, the samples were bisected, and there was only one sample misclassified in our cohort (Supplementary Figure 1D, Supplemental Digital Content 2, http://links.lww.com/JS9/B611). Importantly, the publicly available I-SPY2 dataset containing 29 TNBC patients receiving chemotherapy and immunotherapy was used to validate the accuracy of the model. In this cohort, the difference between pCR and non-pCR patients was significant with an AUC of 0.858 (*P*=0.0011; Figure 5C, D) and an accuracy of 79.3% (Supplementary Figure 1E, Supplemental Digital Content 2, http://links.lww.com/JS9/B611). Presumably, *TAP1* and *IRF4* may be potential predictive biomarkers for response to the NAT in TNBC.

### Dynamic changes of TIME before and after the NAT

By analyzing six paired pCR and three paired non-pCR samples before and after the NAT, we discovered great changes in transcription of pCR samples, including 163 up-regulated genes and 136 down-regulated genes after the NAT (Figure 5E). While the little changes in non-pCR samples were observed, only involving 14 up-regulated genes and 1 down-regulated genes after the NAT (Figure 5F). GSEA further indicated an aggravated hypoxia in non-pCR samples after the NAT (Figure 5G). As shown in Supplementary Figure 2A (Supplemental Digital Content 2, http://links.lww.com/JS9/B611), multiple signaling pathways after the NAT were significantly activated in pCR and non-pCR samples. The proliferation of tumor cells decreased markedly in pCR samples after the NAT, but inflammatory chemokines and endothelial cells increased (Supplementary Figure 2B, Supplemental Digital Content 2, http://links.lww.com/JS9/B611).

## Discussion

In this phase II trial, our results first demonstrated that addition of camrelizumab to nonplatinum-based chemotherapy as the NAT led to a total pCR rate of 65.0% and bpCR rate of 70.0% in patients with untreated, early TNBC, along with a measurable safety profile. In addition, this study also unveiled that *TAP1* and *IRF4* may be the promising predictive biomarkers for response to the NAT, and aggravated hypoxia and activated glycolysis after the NAT may be associated with the treatment resistance.

For locally advanced TNBC, standard anthracycline-taxane-based regimens remain the mainstay of the NAT^17^. Recently, the role of platinum has been gradually realized. In KEYNOTE-522 study, pembrolizumab combined with the chemotherapy of carboplatin followed by anthracycline/cyclophosphamide demonstrated a pCR rate of 64.8% in early TNBC, with known safety profiles of platinum-containing neoadjuvant chemotherapy^3^. Thus, the combination of pembrolizumab with chemotherapy was approved by the US Food and Drug Administration^18^. However, it remains unclear whether the chemotherapy regimen composed of four drugs is necessary or which backbone is the most optimal to enhance the immune response. In this trial, we added camrelizumab to platinum-free chemotherapy regimen for early TNBC and achieved the total pCR rate of 65.0%. Notably, the benefit of axillary downstaging was also derived, with a bpCR rate of 68.7% in patients with positive axillary lymph nodes, higher than previously reported 48.0% using the standard chemotherapy regimen^19^.

In this trial, the BCS rate was 20.0%, significantly lower than the pCR rate, which may be partially explained by the fact that only about 17.4% of patients chose BCS at our center after the NAT^19^, even though ~50% of the patients were suitable^20^. Consistent with previous reports^5,21^, we observed inconsistent responses in radiology and pathology. Notably, there were no prominent changes in tumor burden from the baseline in the efficacy-evaluable population after 5 to 8 cycles of the NAT.

Regarding the AEs, the treatment regimen is well tolerated in our patients. AEs observed in this study were generally consistent with the known safety profiles of neoadjuvant camrelizumab for patients with early TNBC. The most common treatment-related AEs of grade 3 or greater were neutropenia (65.2%), leucopenia (52.2%) and decreased lymphocyte count (17.4%), which is similar to previous reports^22^. In previous meta-analyses, platinum-free neoadjuvant chemotherapy was associated with a lower incidence of grade 3 or grade 4 hematological AEs such as neutropenia and anemia compared to platinum-based chemotherapy^23^.

In this study, the vast majority of immune-related AEs are grade 1 or 2. Two patients (8.7%) experienced grade 4 pneumonia. Reactive cutaneous capillary endothelial proliferation (60.9%) was the most frequent immune-related AEs, followed by hypothyroidism (26.1%), hyperthyroidism (8.7%), pneumonia (8.7%), and more, which is similar to previous reports^22,24^. The mechanism of action and pharmacological differences in chemotherapy drugs may increase the possibility of toxicities^25^. However, caution should be exercised in interpreting indirect cross trial comparisons. Closely monitoring AEs that occur during the treatment process and distinguishing AEs related to immunotherapy are of great significance for clinical patient management.

We discovered that pCR patients had higher scores of T cell markers, antigen presentation machinery and immunoproteasome than non-pCR patients before the NAT, and the differences were pronounced in TIME characteristics, indicating a higher immune infiltration level in pCR patients before the NAT. By establishing a NAT predictive model, we demonstrated that the NISS could predict the NAT efficacy well, with the AUC of 0.967, and this predictive performance had been validated in I-SPY2-TNBC cohort, suggesting *TAP1* and *IRF4* might be the novel predictive biomarkers for responses to the NAT in early TNBC. *TAP1*, a major histocompatibility complex (MHC)-II-encoded gene, is essential to produce cellular immune responses, and its polymorphism can preferentially affect the specificity of peptides transported by MHC class I molecules and the outcome of immune responses^26^. The expression of TAP1 is elevated in multiple tumors, and high expression is positively correlated with immune cell infiltration and poor prognosis^27^. A previous study has confirmed the involvement of TAP1 in anti-PD-1 antibody immunotherapy medicated by IL-27 in small cell lung cancer^28^. IRF4, one member with very limited expression pattern from the family of transcriptional regulators, plays an important role in the function of immune cells and is necessary for CD8+ T cell activation, proliferation, and differentiation to effector cells^29,30^. Thus, the potential prognostic roles of TAP1 and IRF4 in patients receiving the NAT still need to be further explored.

GSEA indicated glycolysis and hypoxia were significantly activated in non-pCR samples before the NAT, and the hypoxia was exacerbated after treatment. Meanwhile, the NISS was identified to be negatively related to hypoxia and glycolysis. A previous study showed that high levels of glycolysis might facilitate tumor immune evasion^31^. In the tumor environment, hypoxia can up-regulate the genes encoding glucose transporters and glycolytic enzymes including lactate dehydrogenase, leading to production and secretion of lactic acid from tumor cells^32^. A large amount of lactic acid and H+ accumulating in the tumor environment can affect the proliferation and survival of infiltrating T cells and generation of cytokines, and damage the immune balance in the tumor environment, which is favorable for tumor immune evasion and progression. Moreover, continuous antigenic stimulation under hypoxia can rapidly and severely promote T cell dysfunction and exhaustion^33^. Accordingly, we speculated that aggravated hypoxia and activated glycolysis after the NAT might have connection with the treatment resistance.

The limitations of the study included single-arm design with small sample sizes, short-term outcomes, and no control group. The proportion of patients with positive lymph nodes was relatively high in the study population. In the future, large-scale randomised studies with long follow-up are needed to determine whether the removal of platinum would not affect the prognosis.

## Conclusion

In conclusion, neoadjuvant camrelizumab combed with nonplatinum-based chemotherapy shows a favorable pCR rate in early TNBC, with an acceptable safety profile. TAP1 and IRF4 may be promising predictive biomarkers for response to the NAT, and aggravated hypoxia and activated glycolysis after the NAT may be associated with the treatment resistance. Future randomized controlled trials are warranted to confirm these findings.

## Ethical approval

The protocol was approved by the Institutional Review Board of Shandong Cancer Hospital and Institute (SDZLEC2020-022-01). The trial was conducted in accordance with the principles of Declaration of Helsinki and Good Clinical Practice guidelines. Written informed consent was provided by each patient before enrollment.

## Consent

Written informed consent was obtained from the patient for publication of this case report and accompanying images. A copy of the written consent is available for review by the Editor-in-Chief of this journal on request.

## Sources of funding

This work was supported by the China Postdoctoral Science Foundation (Grant No. 2022M721988); Wujieping Science Foundation of China (Grant No. 320.6750.2023-18-3); and Natural Science Foundation of Shandong Province (Grant No. ZR2022QH041).

## Author contribution

C.H.Z. and Y.S.W.: participated in the study conception, design, and planning; Y.B.L., Z.B., P.F.Q., Z.Q.S., Z.P.Z., P.C., X.S., and C.J.W.: were responsible for data acquisition; X.E.W., G.D.Q., X.B.,S.G.Z., and X.J.M.: participated in statistical analysis and interpretation of the results; Y.J.S., Y.X.Q., L.L., and N.N.L.: provided technical and material support; C.H.Z.: drafted the manuscript: Y.S.W.: critically revised the manuscript. All authors reviewed the manuscript and agreed to submit it for publication.

## Conflicts of interests disclosure

The authors declare that they have no competing interests.

## Research registration unique identifying number (UIN)

NCT04676997.

## Guarantor

Yongsheng Wang.

## Data availability statement

The datasets generated during and/or analyzed during the current study are available from the corresponding author (Yong-Sheng Wang) on reasonable request. The corresponding author had full access to all the data in the study and had final responsibility for the decision to submit for publication.

## Provenance and peer review

Not commissioned, externally peer-reviewed.

## Supplementary Material

SUPPLEMENTARY MATERIAL
