# Supplementary material for: Clinical efficacy and biomarker analysis of neoadjuvant camrelizumab plus chemotherapy for early-stage triple-negative breast cancer: a experimental single-arm phase II clinical trial pilot study
Source: Int J Surg. 2023 Dec 19;110(3):1527–36. doi: 10.1097/JS9.0000000000001011 (PMC10942181; doi:10.1097/JS9.0000000000001011)
Supplement: SUPPLEMENTARY MATERIAL [file js9-110-1527-s002.docx]

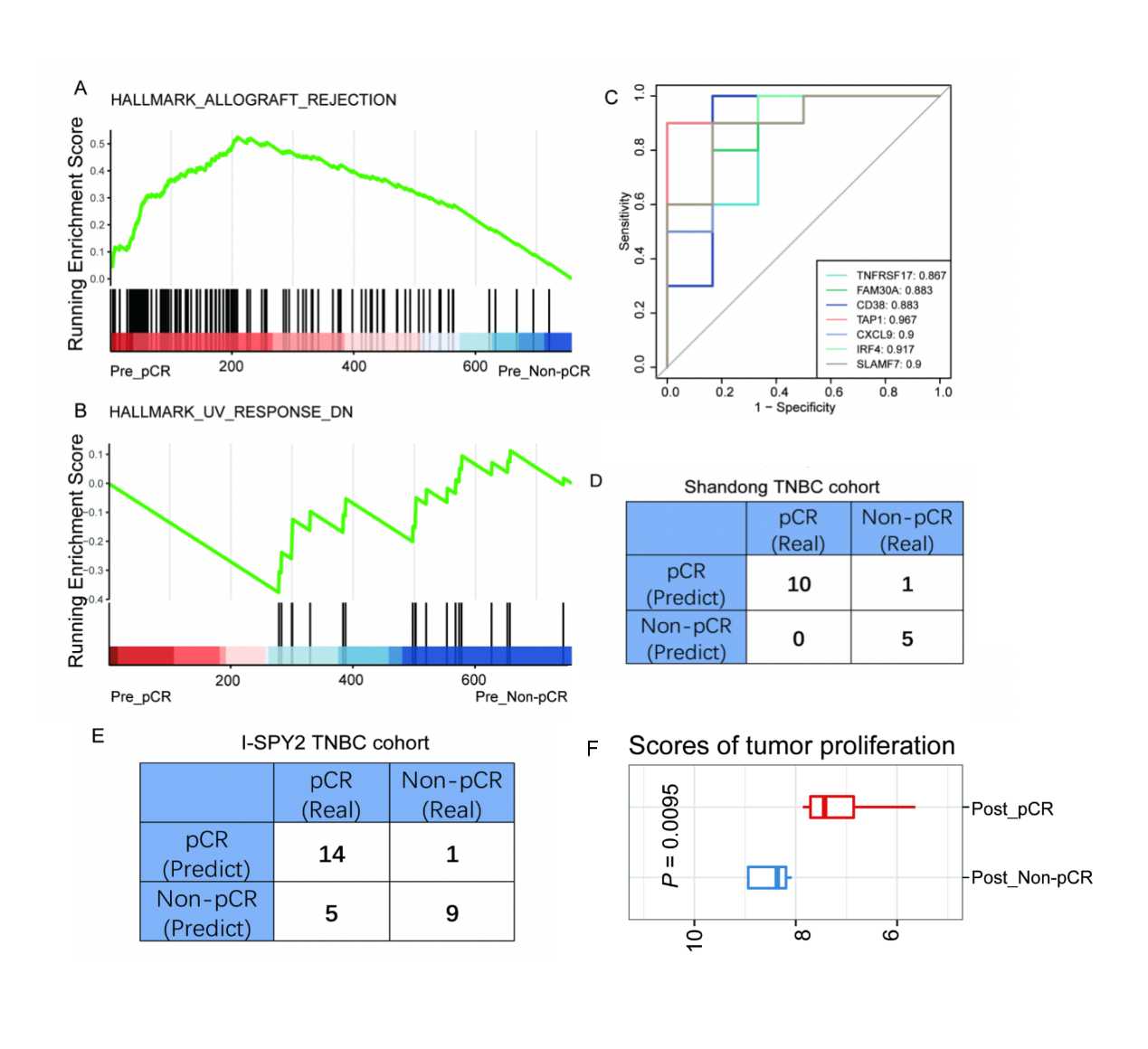


**Supplementary Figure 1.** A-B) Gene set enrichment analysis of the Hallmark allograft rejection and UV response pathways between pCR and non-pCR before the neoadjuvant therapy. C) The receiver operator characteristic curve of 7 single genes in the prediction of response to the neoadjuvant therapy. D-F) The results of samples bisected in our cohort and I-SPY2 TNBC and HR+/HER- cohorts. Pre-treatment pCR: n=10, post-treatment non-pCR: n=6; post-treatment pCR: n=6, post-treatment non-pCR: n=4.


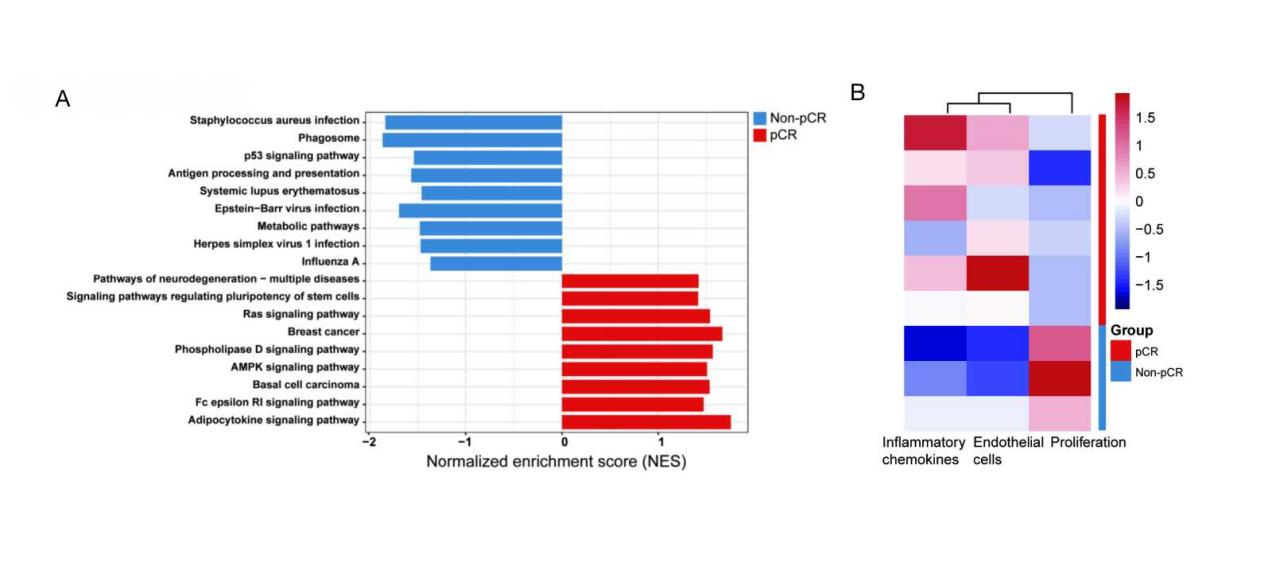


**Supplementary Figure 2.** A-B) Normalized enrichment scores, inflammatory chemokines, endothelial cells, and proliferation between pCR and non-pCR patients after the neoadjuvant therapy.

**Table S1. Comparison of the pre-neoadjuvant baseline information between pCR and non-pCR patients, n (%)**

| Characteristics | Non-pCR (*n*=6) | pCR (*n*=10) | *P* value |
| --- | --- | --- | --- |
| Median age (range), years | 49.33 (11.38) | 50.10 (9.83) | 0.889 |
| T stage |  |  | 0.202 |
| T1 | 0 (0.0) | 4 (40.0) |  |
| T2 | 4 (66.7) | 4 (40.0) |  |
| T3 | 2 (33.3) | 2 (20.0) |  |
| Nodal status, positive | 5 (83.3) | 10 (100.0) | 0.790 |
| Clinical stage, III | 3 (50.0) | 2 (20.0) | 0.486 |
| TNM stage | |  | 0.083 |
| T1N1 | 0 (0.0) | 4 (40.0) |  |
| T2N0 | 1 (16.7) | 0 (0.0) |  |
| T2N1 | 1 (16.7) | 4 (40.0) |  |
| T2N2 | 2 (33.3) | 0 (0.0) |  |
| T3N1 | 1 (16.7) | 2 (20.0) |  |
| T3N2 | 1 (16.7) | 0 (0.0) |  |

**Abbreviations:** pCR, pathological complete response.
